# Real-world use of over-the-counter medications by patients with migraine in Japan: results from the OVERCOME (Japan) 2nd study

**DOI:** 10.1186/s10194-025-02046-8

**Published:** 2025-05-07

**Authors:** Ryotaro Ishii, Tsubasa Takizawa, Shiho Suzuki, Daisuke Danno, Moemi Miura, Yoshinori Tanizawa, Satoshi Osaga, Chie Hashimoto, Mika Komori

**Affiliations:** 1 Kyoto Prefectural University of Medicine, Kyoto, Japan; 2 Keio University School of Medicine, Tokyo, Japan; 3 Dokkyo Medical University, Mibu, Japan; 4 Tominaga Hospital, Osaka, Japan; 5 Social Survey Research Information Co., Ltd, Tokyo, Japan; 6 Eli Lilly Japan K.K, Kobe, Japan

**Keywords:** Over-the-counter, Survey, Headache, Acute medication, Preventive medication

## Abstract

**Background:**

People with migraine may prefer over-the-counter (OTC) drugs because of multiple reasons, but their overuse can cause medication overuse headaches. This analysis of the ObserVational survey of the Epidemiology, tReatment, and Care Of MigrainE (OVERCOME [Japan]) 2nd study describes the real-world use of OTC headache drugs and the challenges that potentially prevent people with migraine from accessing appropriate medical management in Japan.

**Methods:**

This analysis of the cross-sectional, population-based, nationwide online survey included adults with migraine. Respondents reported their experiences with prescription and OTC drugs for migraine, migraine-specific drug awareness, and attitude towards migraine. Subgroup analyses were performed based on the number of monthly headache days (MHD) and the frequency of OTC drug use/month.

**Results:**

The 19,590 respondents with migraine (68.8% female; mean [SD] age 40.5 [13.1] years) had mean (SD) 3.5 (5.2) MHDs; 29.0% consulted doctors in the past year for migraine. OTC drug use in the past year was common (≥ 62.1%) regardless of doctor consultation or number of MHDs. Among respondents who answered that they would usually use prescribed drugs when they have a migraine attack, 35.2% reported that they would typically use OTC drugs too. The frequency of OTC drug use was the same or higher than that of prescribed drugs in 51.3% of the respondents who consulted doctors in the past year. Only 14.6% of respondents discussed OTC drugs with doctors during consultations in the past year. Migraine-specific drug access and awareness were limited even among frequent OTC drug users (≥ 10 days/month); 18.2% used triptans, but 65.5% never heard of it. Among 37.1% of respondents who had hesitated to visit a doctor, ‘I could handle it myself with OTC medicine’ was the most common reason for hesitation (34.9%).

**Conclusion:**

OTC drug use is common among people with migraine; however, it is not frequently discussed with doctors. Many respondents, even those with frequent OTC drug use, did not have access or awareness of migraine-specific drugs. To prevent medication overuse for migraine, the use of OTC drugs should also be discussed and managed.

**Supplementary Information:**

The online version contains supplementary material available at 10.1186/s10194-025-02046-8.

## Introduction

Migraine is a chronic neurologic disease with a prevalence rate of 7.3–8.4% in Japan [1, 2]. The 2021 Japanese headache guidelines recommend acute treatments for migraine, including over-the-counter (OTC) medications such as acetaminophen or non-steroidal anti-inflammatory drugs (NSAIDs) and prophylactic therapy [3]. However, as reported in the ObserVational survey of the Epidemiology, tReatment, and Care Of MigrainE (OVERCOME [Japan]), 42.6% of patients with migraine do not have adequate access to medical care and appropriate treatment [4].

Patients with migraine may prefer OTC drugs because they are economical and readily available [5], current prescribed drugs result in unsatisfactory outcomes [6], or due to insufficient advanced neurological and headache care and a lack of awareness of proper headache treatment [7]. However, overuse of medications, including OTC drugs, may lead to medication overuse headache (MOH) [8]. The International Classification of Headache Disorders, 3rd edition (ICHD-3) defines MOH as a headache occurring ≥ 15 days/month in individuals with a pre-existing primary headache, developing due to regular overuse of acute or symptomatic headache medications (≥ 10 or ≥ 15 days/month depending on the medication) for > 3 months [9]. In Japan, 2.3–3.7% of patients with migraine may be at risk of MOH [7, 10].

In the real world, clinicians can only understand patients’ OTC drug usage via consultations [11, 12]. However, no matter how clinicians try to capture the exact OTC usage of the patients, there could be limitations as the data are ‘self-reported’. Additionally, OTC drug use is difficult to assess via commonly used real-world data sources such as administrative claims databases because those cannot capture OTC drugs [10]. Therefore, the characteristics, attitudes, and experiences of Japanese patients with migraine who tend to rely on OTC drugs are not well understood.

The OVERCOME (Japan) 2nd study was conducted in 2023, after the approval of new migraine medications in Japan [13], to describe the clinical history, migraine burden, patient-reported outcomes (PRO), and experiences of patients with migraine. This analysis of the OVERCOME (Japan) 2nd study describes the real-world use of OTC drugs among patients with migraine and the challenges that potentially prevent patients from accessing appropriate medical management.

## Methods

### Study design

Danno et al. [14] explicitly describe the study design of the OVERCOME (Japan) 2nd study. In brief, this observational study was conducted via a cross-sectional, population-based, nationwide online survey in adults with and without migraine. The participants were recruited between June 2023 and August 2023 using survey panels from multiple providers in Japan. After screening, eligible participants were divided into a migraine and non-migraine group. The current analysis was only performed among participants with migraine.

The Medical Corporation TOUKEIKAI Kitamachi Clinic ethics committee approved this study (number BGQ09531) on April 19, 2023. The study was conducted per ethical principles originating from the Declaration of Helsinki and was consistent with Good Pharmacoepidemiology Practices. All applicable Japanese laws and regulations were followed. All survey respondents provided electronic informed consent and agreed to participate in the study. All data were anonymized before analysis. The datasets generated and/or analyzed during the current study are available at Eli Lilly Japan K.K on reasonable request.

### Study population and selection criteria

As described previously [14], all participants were ≥ 18 years of age, Japanese residents, had experienced headaches or migraine in the past year, and either self-reported physician-diagnosed migraine or met modified criteria for migraine per International Classification of Headache Disorders – 3rd edition (ICHD-3) criteria [9]. These criteria were validated in the American Migraine Study [15] and the American Migraine Prevalence and Prevention Study [16], as stated in previous OVERCOME studies from Japan [4], the European Union (EU) [17], and the United States (US) [18]. Participants were excluded if all headaches experienced in the past year were secondary headaches caused by hangovers, infections, or trauma; or if inconsistencies were present between the answers to gender and disease.

### Variables and outcomes

Participants reported demographic data such as age and sex, clinical characteristics such as the average number of monthly headache days (MHD), the occurrence of probable MOH per ICHD-3 criteria [9] within the past 90 days, and physician consultations over the past year. The impact of migraine was assessed using PRO instruments such as the Japanese Migraine Disability Assessment Scale (MIDAS) questionnaire [19], the Japanese version of the Headache Impact Test-6 (HIT-6) [20], and Migraine Interictal Burden Scale-4 (MIBS-4) [21, 22] (Additional file 1).

Participants also reported their experiences with past and current use of prescription and OTC drugs for migraine, specifically the medications they usually took for migraine attacks. The assessment included monthly medication usage over the past 90 days, any combinations used, triggers for using OTC drugs, awareness about migraine-specific medications, and their attitude towards migraine. Additional file 2 lists the OTC drugs available in Japan for migraine treatment.

### Statistical analysis

Data were summarized with descriptive statistics: means and standard deviations (SDs) reported for continuous variables, and frequencies and percentages reported for categorical variables. Subgroup analyses were performed to describe: (a) differences in respondent characteristics and medication use based on the number of MHDs (0–3 MHD, 4–7 MHD, 8–14 MHD, and ≥ 15 MHD i.e. chronic migraine [CM; operational definition based on the data obtainable in the survey]); and (b) differences in respondent characteristics, medical management of migraine, and patient attitudes based on the number of monthly days of OTC drug use (0, 1–4, 5–9, and ≥ 10).

The results were not adjusted for bias and confounding, and statistical comparisons were not performed. Descriptive statistics for categorical and continuous variables were obtained with Python version 3.9.7 (Python Software Foundation) and BellCurve for Excel version 4.04 or later (Social Survey Research Information Co., Ltd., Tokyo, Japan), respectively.

## Results

**Fig. 1:**
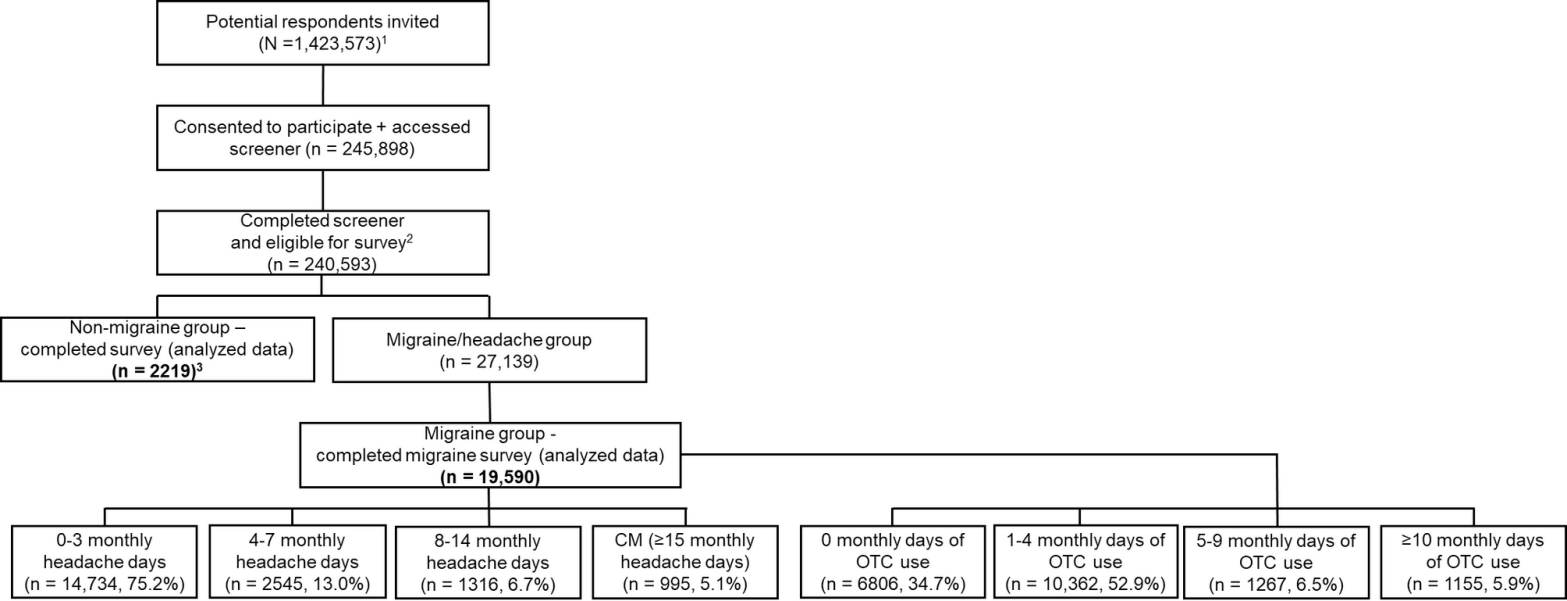
Study participant flow chart ^1^ Targeted sampling to represent the Japanese adult population in terms of key demographic characteristics (age, sex, and geography) was applied. ^2^ This included those who passed the screener stage and represented the Japanese census adult population. ^3^ A quota of 2000 was set for the non-migraine group, and about 2000 respondents were selected from the eligible population while preserving its demographic composition. The majority of respondents eligible for this group were over quota. Abbreviations: CM – Chronic Migraine, N – Total population size, n – Sample size, OTC – Over-the-counter

Among the 240,593 eligible respondents for the survey, final analyses were conducted on 19,590 respondents with migraine (Fig. 1). The respondents were stratified into subgroups based on the number of MHDs: 0–3 MHD (*n* = 14,734), 4–7 MHD (*n* = 2545), 8–14 MHD (*n* = 1316), and CM (*n* = 995); and the number of monthly days with OTC use: 0 days (*n* = 6806), 1–4 days (*n* = 10,362), 5–9 days (*n* = 1267), and ≥ 10 days (*n* = 1155).

### Demographic characteristics, clinical characteristics, and patient-reported outcomes

The mean (SD) respondent age was 40.5 (13.1) years, and 68.8% were females (Table 1). The majority of respondents met ICHD-3 criteria for migraine (84.9%), but less than half (46.4%) self-reported a physician migraine diagnosis. Respondents experienced a mean (SD) of 3.5 (5.2) MHD at the time of the survey, but only 29.0% (*n* = 5684) consulted doctors for migraine in the past year. The burden of migraine described in Table 1 has also been reported earlier [14]. Among respondents with probable MOH (2.6%) (Table 1), combination analgesics-overuse headache (56.5%) and NSAIDs-overuse headache (34.6%) were the most common MOH subtypes (Additional file 3).

**Table 1:** Demographic characteristics, clinical characteristics, and patient-reported outcomes^1^

	Total (*N* = 19,590)	0–3 MHD (*n* = 14,734)	4–7 MHD (*n* = 2545)	8–14 MHD (*n* = 1316)	CM, ≥ 15 MHD (*n* = 995)
**Age (years)**,** mean (SD)**	40.5 (13.1)	40.5 (13.3)	40.3 (12.6)	40.3 (12.5)	41.8 (12.8)
**Female**,** n (%)**	13,486 (68.8)	9977 (67.7)	1838 (72.2)	952 (72.3)	719 (72.3)
**Married**,** n (%)**	8881 (45.3)	6755 (45.8)	1175 (46.2)	559 (42.5)	392 (39.4)
**Employed**,** n (%)**	12,724 (65.0)	9617 (65.3)	1663 (65.3)	841 (63.9)	603 (60.6)
**Diagnosis of migraine by a physician**,** n (%)**	9081 (46.4)	6578 (44.6)	1254 (49.3)	714 (54.3)	535 (53.8)
Age at migraine diagnosis, mean (SD)	26.9 (11.4)	26.7 (11.4)	27.7 (11.2)	27.9 (11.3)	26.8 (11.9)
**Met ICHD-3 criteria**	16,627 (84.9)	12,261 (83.2)	2282 (89.7)	1176 (89.4)	908 (91.3)
Met ICHD-3 criteria and diagnosed as migraine	6118 (31.2)	4105 (27.9)	991 (38.9)	574 (43.6)	448 (45.0)
**Average MHDs in the past 90 days**,** mean (SD)**	3.5 (5.2)	1.4 (1.0)	5.5 (1.0)	10.3 (1.6)	21.9 (5.5)
**Pain severity**,** mean (SD)**	5.7 (2.0)	5.5 (2.1)	6.3 (1.6)	6.5 (1.6)	7.0 (1.7)
**MIDAS score**,** mean (SD)**^**2**^	10.0 (20.9)	6.4 (13.9)	14.4 (20.2)	22.4 (29.8)	38.6 (49.2)
**MIDAS grade**,** n (%)**^**2**^					
Grade I (0–5)	12,110 (62.2)​	10,291 (70.0)	1036 (40.9)	477 (36.5)	306 (32.6)
Grade II (6–10)	2659 (13.7)​	2027 (13.8)	413 (16.3)	141 (10.8)	78 (8.3)
Grade III (11–20)	2220 (11.4)​	1365 (9.3)	529 (20.9)	223 (17.1)	103 (11.0)
Grade IV (21+)	2485 (12.8)​	1014 (6.9)	553 (21.8)	466 (35.7)	452 (48.1)
**HIT-6 score**,** mean (SD)**	59.7 (7.8)	58.5 (7.7)	62.4 (6.4)	63.5 (6.7)	65.7 (7.3)
**HIT-6 grade**,** n (%)**					
Little-to-no impact (36–49)	2004 (10.2)	1885 (12.8)	66 (2.6)	35 (2.7)	18 (1.8)
Moderate impact (50–55)	3196 (16.3)	2823 (19.2)	225 (8.8)	88 (6.7)	60 (6.0)
Substantial impact (56–59)	3283 (16.8)	2613 (17.7)	411 (16.1)	181 (13.8)	78 (7.8)
Severe impact (60–78)	11,107 (56.7)	7413 (50.3)	1843 (72.4)	1012 (76.9)	839 (84.3)
**MIBS-4 score**,** mean (SD)**	3.2 (3.6)	2.9 (3.5)	3.8 (3.7)	4.2 (3.9)	5.1 (4.1)
**MIBS-4 level of interictal burden**,** n (%)**					
None (0)	8216 (41.9)	6681 (45.3)	880 (34.6)	409 (31.1)	246 (24.7)
Mild (1–2)	2152 (11.0)	1602 (10.9)	320 (12.6)	143 (10.9)	87 (8.7)
Moderate (3–4)	2886 (14.7)	2191 (14.9)	370 (14.5)	185 (14.1)	140 (14.1)
Severe (5+)	6336 (32.3)	4260 (28.9)	975 (38.3)	579 (44.0)	522 (52.5)
**Ever visited a doctor**,** n (%)**	11,758 (60.0)	8465 (57.5)	1669 (65.6)	908 (69.0)	716 (72.0)
Over the past year	5684 (29.0)	3788 (25.7)	929 (36.5)	537 (40.8)	430 (43.2)
**Probable MOH**,** n (%)**	503 (2.6)	0 (0.0)	0 (0.0)	0 (0.0)	503 (50.6)

^1^ Most of the data were previously reported [14]. ^2^ Sample sizes for the MHD subgroups were: 0–3 = 14,697, 4–7 = 2531, 8–14 = 1307, and ≥ 15 = 939. During data cleaning, respondents with inappropriate responses for MIDAS questions, i.e., > 90 days of disability due to migraine in a 90-day period, were excluded. Abbreviations: CM – Chronic Migraine, HIT-6 – Headache Impact Test-6, ICHD-3 - International Classification of Headache Disorders – 3rd edition, MHD – Monthly Headache Days, MIBS – Migraine Interictal Burden Scale-4, MIDAS – Migraine Disability Assessment Scale, MOH – Medication Overuse Headache, N – Total population size, n – Sample size, OTC – Over-the-counter, SD – Standard Deviation

The migraine subgroups (0–3 MHD, 4–7 MHD, 8–14 MHD, and CM) had generally similar mean age and gender distribution (Table 1). The 8–14 MHD and CM subgroups had high proportions of respondents with a self-reported physician migraine diagnosis (≥ 53.8%). In the MHD subgroups, an increase in the number of MHDs showed a trend of greater respondent burden and doctor visits.

Subgroup analysis by frequency of OTC drug use showed that respondents with ≥ 5 monthly days of OTC drug use had numerically more MHDs and greater migraine-related burden than respondents with ≤ 4 monthly days of OTC drug use (Additional file 4). The ≥ 10 monthly days of OTC drug use subgroup had the highest proportion of respondents with probable MOH (29.6%).

### OTC and prescription drug use

The majority of the respondents had ever used OTC (84.4%) or prescribed acute drugs (56.6%) in the past, but preventive drug use was low (25.7%) (Table 2). Prescribed acute drug use in the past year and current preventive drug use were numerically greater among respondents who consulted doctors for migraine in the past year (83.2% and 36.0%) compared with the overall population (42.6% and 13.4%). On the other hand, OTC drug use in the past year was numerically lower in this subpopulation compared with the overall population (65.6% vs. 72.7%). An increase in the number of MHDs showed a trend of greater use of prescribed acute and preventive drugs in the overall population and subpopulation who consulted doctors for migraine in the past year (Table 2).

**Table 2:** Prevalence of OTC and prescription drug use

		Overall respondents with migraine										Respondents who consulted doctors for migraine in the past year								
		Total (*N* = 19,590)		0–3 MHD (*n* = 14,734)		4–7 MHD (*n* = 2545)		8–14 MHD (*n* = 1316)		CM, ≥ 15 MHD (*n* = 995)		Total (*n* = 5684)		0–3 MHD (*n* = 3788)		4–7 MHD (*n* = 929)	8–14 MHD (*n* = 537)		CM, ≥ 15 MHD (*n* = 430)	
**Ever used prescribed acute drugs**,** n (%)**		11,082 (56.6)		7866 (53.4)		1634 (64.2)		897 (68.2)		685 (68.8)		5221 (91.9)		3417 (90.2)		886 (95.4)	507 (94.4)		411 (95.6)	
**Ever used OTC medications**,** n (%)**		16,526 (84.4)		12,306 (83.5)		2255 (88.6)		1143 (86.9)		822 (82.6)		4688 (82.5)		3115 (82.2)		784 (84.4)	451 (84.0)		338 (78.6)	
**Ever used preventive drugs**,** n (%)**		5038 (25.7)		3450 (23.4)		790 (31.0)		437 (33.2)		361 (36.3)		3152 (55.5)		2028 (53.5)		557 (60.0)	304 (56.6)		263 (61.2)	
**Used prescribed acute drugs in the past year**,** n (%)**		8344 (42.6)		5777 (39.2)		1281 (50.3)		726 (55.2)		560 (56.3)		4730 (83.2)		3047 (80.4)		816 (87.8)	479 (89.2)		388 (90.2)	
NSAIDs		5299 (27.0)		3604 (24.5)		821 (32.3)		494 (37.5)		380 (38.2)		2802 (49.3)		1746 (46.1)		485 (52.2)	314 (58.5)		257 (59.8)	
Acetaminophen		1657 (8.5)		1145 (7.8)		265 (10.4)		120 (9.1)		127 (12.8)		845 (14.9)		543 (14.3)		146 (15.7)	66 (12.3)		90 (20.9)	
Triptans		1731 (8.8)		1099 (7.5)		308 (12.1)		194 (14.7)		130 (13.1)		1516 (26.7)		935 (24.7)		287 (30.9)	175 (32.6)		119 (27.7)	
**Used OTC drugs in the past year**,** n (%)**		14,234 (72.7)		10,545 (71.6)		1991 (78.2)		994 (75.5)		704 (70.8)		3729 (65.6)		2492 (65.8)		612 (65.9)	358 (66.7)		267 (62.1)	
Combination analgesics		9330 (47.6)		6715 (45.6)		1402 (55.1)		698 (53.0)		515 (51.8)		2489 (43.8)		1615 (42.6)		437 (47.0)	252 (46.9)		185 (43.0)	
NSAIDs		7490 (38.2)		5577 (37.9)		1050 (41.3)		530 (40.3)		333 (33.5)		2127 (37.4)		1407 (37.1)		361 (38.9)	204 (38.0)		155 (36.0)	
Acetaminophen		547 (2.8)		390 (2.6)		89 (3.5)		34 (2.6)		34 (3.4)		244 (4.3)		159 (4.2)		44 (4.7)	20 (3.7)		21 (4.9)	
**Current use of preventive drugs**,** n (%)**		2628 (13.4)		1740 (11.8)		411 (16.1)		255 (19.4)		222 (22.3)		2047 (36.0)		1290 (34.1)		357 (38.4)	211 (39.3)		189 (44.0)	
Oral		2508 (12.8)		1670 (11.3)		386 (15.2)		242 (18.4)		210 (21.1)		1944 (34.2)		1233 (32.6)		335 (36.1)	198 (36.9)		178 (41.4)	
CGRP mAbs		188 (1.0)		102 (0.7)		39 (1.5)		23 (1.7)		24 (2.4)		177 (3.1)		96 (2.5)		36 (3.9)	23 (4.3)		22 (5.1)	
Other injectables		120 (0.6)		84 (0.6)		14 (0.6)		10 (0.8)		12 (1.2)		106 (1.9)		72 (1.9)		13 (1.4)	10 (1.9)		11 (2.6)	
**Respondents’ awareness of migraine-specific drugs, n (%)**																				
Never heard of any triptan		14,316 (73.1)		11,041 (74.9)		1739 (68.3)		857 (65.1)		679 (68.2)		2639 (46.4)		1816 (47.9)		394 (42.4)	231 (43.0)		198 (46.0)	
Never heard of lasmiditan		17,612 (89.9)		13,289 (90.2)		2266 (89.0)		1175 (89.3)		882 (88.6)		4432 (78.0)		2933 (77.4)		722 (77.7)	437 (81.4)		340 (79.1)	
Never heard of any CGRP mAbs		17,524 (89.5)		13,248 (89.9)		2245 (88.2)		1154 (87.7)		877 (88.1)		4344 (76.4)		2882 (76.1)		714 (76.9)	417 (77.7)		331 (77.0)	

Abbreviations: CGRP mAbs - Calcitonin Gene-related Peptide Monoclonal Antibodies, CM – Chronic Migraine, MHD – Monthly Headache Days, N – Total population size, n – Sample size, NSAID – Non-steroidal Anti-inflammatory Drug, OTC – Over-the-counter

**Fig. 2:**
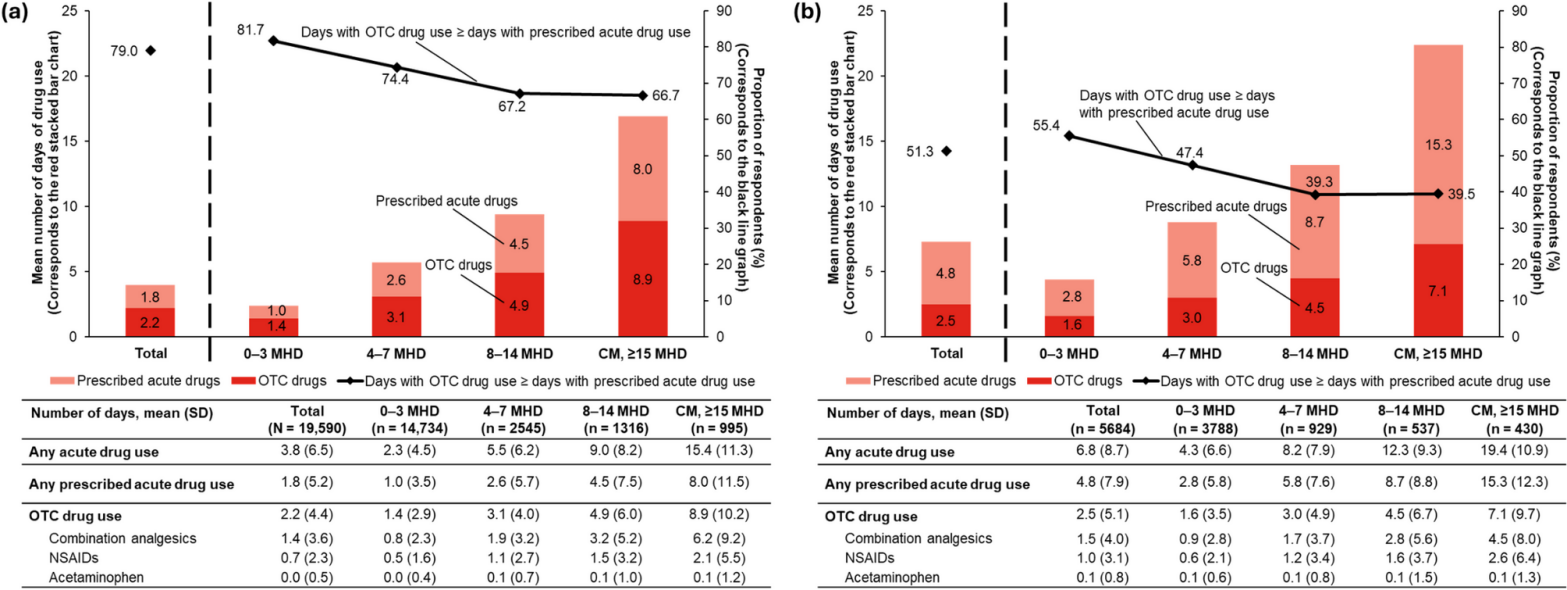
Monthly use of prescribed acute and OTC drugs. (**a**) Overall respondents with migraine and (**b**) Respondents who consulted doctors for migraine in the past year. The stacked bar chart corresponds to the left Y-axis and the black line graph corresponds to the right Y-axis. In the survey, respondents answered ‘number of days of drug use’ separately for each individual drug. The counts were summed up to obtain the total reported here for each of the summed number of days of any prescribed acute drug use and summed number of days of any OTC drug use. We reported this in the table, as well as in the stacked bar chart. The respondents also separately answered the number of days of any acute drug use (including both OTC and prescribed drugs); this is only shown in the table below the graph. The sum of number of days of ‘OTC drug use’ and number of days of ‘any prescribed acute drug use’ is not equal to ‘any acute drug use’, because multiple drugs may have been used in combination on the same day. Abbreviations: CM – Chronic Migraine, MHD – Monthly Headache Days, N – Total population size, n – Sample size, NSAID – Non-steroidal Anti-inflammatory Drug, OTC – Over-the-counter, SD – Standard Deviation

The frequency of acute drug use in total, including both prescribed and OTC drugs, was numerically greater among respondents who consulted doctors for migraine in the past year than in the overall population (mean [SD] number of days/month 6.8 [8.7] vs. 3.8 [6.5]) (Fig. 2a and b). The frequency of prescribed acute drug use showed the same trend (4.8 [7.9] vs. 1.8 [5.2]). On the other hand, the frequency of OTC drug use was similar among respondents who consulted doctors for migraine in the past year and the overall population (mean [SD] number of days/month 2.5 [5.1] vs. 2.2 [4.4]). The proportion of respondents whose frequency of OTC drug use was equal to or more than that of prescribed acute drug use was lower in respondents who consulted doctors in the past year than in the overall population (51.3% vs. 79.0%) (Fig. 2a and b).

The average frequency of any acute, prescribed acute, and OTC drug use/month numerically increased with greater MHD subgroups in the overall population and the subpopulation who consulted doctors for migraines in the past year (Fig. 2a and b). The proportion of respondents whose frequency of OTC drug use was equal to or more than that of prescribed acute drug use was highest in the 0–3 MHD subgroup (81.7% and 55.4%). This proportion was 66.7% and 39.5% in the CM subgroups of the overall population and subpopulation, respectively.

The survey also asked respondents what acute drugs they would usually use when they had migraine attacks. Here too, respondents who consulted doctors for migraines in the past year would usually use prescribed acute drugs more than the overall population (62.3% vs. 32.7%) (Table 3). The use of each prescribed drug class, namely triptans, NSAIDs, and acetaminophen, showed the same trend. On the other hand, the proportion of OTC drug use was numerically lower in those who consulted doctors in the past year compared with the overall population (53.6% vs. 75.2%).

**Table 3:** Acute OTC and prescription medications respondents would usually use when they had migraine attacks^1^

	Overall respondents with migraine	Respondents who consulted doctors for migraine in the past year
	Total (*n* = 17,094)^2^	Total (*n* = 5449)^2^
**Any OTC drug**,** n (%)**	12,858 (75.2)	2923 (53.6)
**Any prescribed acute drug**,** n (%)**	5591 (32.7)	3394 (62.3)
Any prescribed acute drug + any OTC drug^3^	1969 (35.2)	1063 (31.3)
**Any triptan**,** n (%)**	1001 (5.9)	899 (16.5)
Any triptan + any OTC drug^4^	271 (27.1)	236 (26.3)
**Any prescribed NSAID**,** n (%)**	3159 (18.5)	1757 (32.2)
Any prescribed NSAID + any OTC drug containing NSAIDs^5,6^	1261 (39.9)	637 (36.3)
**Any prescribed acetaminophen**,** n (%)**	933 (5.5)	494 (9.1)
Any prescribed acetaminophen + any OTC drug containing acetaminophen^5,7^	103 (11.0)	67 (13.6)

^1^ Respondents answered this question in the survey - “Please choose the medications you usually take for pain or during a headache attack, when you have a migraine/headache attack. If you use a combination of two or more medications, please check multiple options.” ^2^ Respondents who used any acute medication in the past year. ^3^ Proportion calculated from the number of respondents on any prescribed acute drug i.e., 5591 or 3394, respectively. ^4^ Proportion calculated from the number of respondents on any triptan i.e., 1001 or 899, respectively. ^5^ Includes combination analgesics. ^6^ Proportion calculated from the number of respondents on any prescribed NSAID i.e., 3159 or 1757, respectively. ^7^ Proportion calculated from the number of respondents on any prescribed acetaminophen i.e., 933 or 494, respectively

Abbreviations: n – Sample size, NSAID – Non-steroidal Anti-inflammatory Drug, OTC – Over-the-counter

Among respondents in the overall population who would usually use prescribed acute drugs for their migraine attacks, 35.2% answered that they would also use OTC drugs (Table 3). This proportion was similar (31.3%) in the subpopulation who consulted doctors in the past year. When evaluated by each class of prescribed acute drug that the respondents would usually use, the use of any OTC drug in addition to a triptan (27.1% and 26.3%), an NSAID-containing OTC drug in addition to a prescribed NSAID (39.9% and 36.3%), and an acetaminophen-containing OTC drug in addition to a prescribed acetaminophen (11.0% and 13.6%), was similar in the overall population and the subpopulation who consulted doctors in the past year, respectively.

### Respondents’ medical management, experience, and awareness of migraine-specific drugs

Most respondents (74.3%) never used preventive drugs for migraine, while 43.4% never used prescribed acute drugs (Table 4). Herbal medicine (9.4%) and NSAIDs or acetaminophen (42.9%) were the most used preventive and prescribed acute drugs, respectively. Use of migraine-specific drugs (calcitonin gene-related peptide monoclonal antibodies [CGRP mAbs] and ditans [both 2.2%], and triptans [13.5%]) was low.

**Table 4:** Medical management of migraine by monthly days of OTC drug use

	Total (*N* = 19,590)	0 days (*n* = 6806)	1–4 days (*n* = 10,362)	5–9 days (*n* = 1267)	≥ 10 days (*n* = 1155)
**Ever visited a doctor**,** n (%)**	11,758 (60.0)	4178 (61.4)	5925 (57.2)	833 (65.7)	822 (71.2)
Over the past year	5684 (29.0)	2355 (34.6)	2508 (24.2)	389 (30.7)	432 (37.4)
**Prescription drug use for migraine**,** n (%)**					
**Preventive drugs**					
Herbal medicine	1843 (9.4)	613 (9.0)	902 (8.7)	148 (11.7)	180 (15.6)
Other orals	1376 (7.0)	574 (8.4)	604 (5.8)	95 (7.5)	103 (8.9)
Calcium channel blockers	1127 (5.8)	442 (6.5)	476 (4.6)	102 (8.1)	107 (9.3)
Beta-blocker	1006 (5.1)	343 (5.0)	490 (4.7)	80 (6.3)	93 (8.1)
Anticonvulsant	893 (4.6)	329 (4.8)	378 (3.6)	80 (6.3)	106 (9.2)
Antidepressant	473 (2.4)	168 (2.5)	193 (1.9)	51 (4.0)	61 (5.3)
Other injectables	456 (2.3)	150 (2.2)	213 (2.1)	34 (2.7)	59 (5.1)
CGRP mAbs	422 (2.2)	121 (1.8)	184 (1.8)	47 (3.7)	70 (6.1)
Have not used preventive medications	14,552 (74.3)	4854 (71.3)	8042 (77.6)	896 (70.7)	760 (65.8)
**Prescribed acute drugs**					
NSAIDs or acetaminophen	8404 (42.9)	2930 (43.1)	4276 (41.3)	616 (48.6)	582 (50.4)
Triptan	2636 (13.5)	1148 (16.9)	1082 (10.4)	196 (15.5)	210 (18.2)
Herbal medicine	1794 (9.2)	616 (9.1)	847 (8.2)	139 (11.0)	192 (16.6)
Antiemetics	1423 (7.3)	539 (7.9)	605 (5.8)	102 (8.1)	177 (15.3)
Other	1136 (5.8)	503 (7.4)	466 (4.5)	66 (5.2)	101 (8.7)
Opioid	482 (2.5)	162 (2.4)	201 (1.9)	39 (3.1)	80 (6.9)
Ditan	440 (2.2)	227 (3.3)	151 (1.5)	25 (2.0)	37 (3.2)
Ergotamine	229 (1.2)	74 (1.1)	95 (0.9)	25 (2.0)	35 (3.0)
Have not used prescribed acute drugs	8508 (43.4)	2640 (38.8)	4954 (47.8)	502 (39.6)	412 (35.7)
**Respondents’ awareness of migraine-specific drugs**,** n (%)**					
Never heard of any triptan	14,316 (73.1)	4817 (70.8)	7862 (75.9)	880 (69.5)	757 (65.5)
Never heard of lasmiditan	17,612 (89.9)	6108 (89.7)	9406 (90.8)	1096 (86.5)	1002 (86.8)
Never heard of any CGRP mAbs	17,524 (89.5)	6108 (89.7)	9325 (90.0)	1101 (86.9)	990 (85.7)

Abbreviations: CGRP mAbs - Calcitonin Gene-related Peptide Monoclonal Antibodies, N – Total population size, n – Sample size, NSAID – Non-steroidal Anti-inflammatory Drug, OTC – Over-the-counter

Subgroup analysis by frequency of OTC drug use showed that, even among respondents with ≥ 10 monthly days of OTC drug use, as many as 28.8% had never visited a doctor (Table 4). Moreover, 65.8% and 35.7% of respondents in this subgroup never used preventive and prescribed acute drugs, respectively. Use of CGRP mAbs increased with greater frequency of OTC drug use, but no clear trend was observed in triptan and ditan usage.

The majority of the respondents (73.1–89.9%) had never heard of migraine-specific drugs (triptans, CGRP mAbs, and lasmiditan) (Table 4). Awareness of these drugs was numerically higher among respondents with ≥ 5 monthly days of OTC drug use (13.1–34.5%) than in those with ≤ 4 monthly days of OTC drug use (9.2–29.2%). Among the respondents who consulted doctors for migraine in the past year, 46.4% were not aware of triptans, but the majority had never heard of CGRP mAbs (76.4%) or lasmiditan (78.0%; Table 2).

### Respondents’ communication with doctors and attitude toward medical consultation for migraine

Among respondents who had experienced consultations with a doctor for their headaches, 37.1% had ever hesitated in the past to visit a doctor (Table 5). This proportion tended to increase with greater frequency of OTC drug use. ‘I could handle it myself with OTC headache medicine’ was the most common reason for hesitating to visit a doctor (34.9%), even among respondents with ≥ 10 monthly days of OTC drug use (44.5%) (Table 5). Even though respondents with ≥ 10 monthly days of OTC drug use had significant migraine burden (mean [SD] MHD 11.5 [9.0]; Additional file 4), approximately 30% of these respondents responded, ‘Even after consulting, I was only prescribed medication similar to OTC headache medicine’ and ‘I thought there wouldn’t be better treatments or remedies than what I was already using.’

**Table 5:** Respondents’ hesitation to visit Doctors (by monthly days of OTC use)

	Total (*n* = 8516)^1^	0 days (*n* = 3140)	1–4 days (*n* = 4089)	5–9 days (*n* = 640)	≥ 10 days (*n* = 647)
**Ever hesitated to visit a doctor**, ***n*** (%)					
Yes	3159 (37.1)	893 (28.4)	1647 (40.3)	309 (48.3)	310 (47.9)
No	4014 (47.1)	1670 (53.2)	1842 (45.0)	246 (38.4)	256 (39.6)
Don’t remember	1343 (15.8)	577 (18.4)	600 (14.7)	85 (13.3)	81 (12.5)
**Reasons for hesitation**,** n (%)**	**Total** (***n*** = **3159**)^**2**^	**0 days** (***n*** = **893**)	**1–4 days** (***n*** = **1647**)	**5–9 days** (***n*** = **309**)	**≥ 10 days** (***n*** = **310**)
I could handle it myself with OTC headache medicine	1103 (34.9)	173 (19.4)	647 (39.3)**	145 (46.9)**	138 (44.5)**
It felt more like a physical trait than a disease	969 (30.7)	238 (26.7)	494 (30.0)	128 (41.4)**	109 (35.2)**
Everyone has headaches	880 (27.9)	226 (25.3)	476 (28.9)	88 (28.5)	90 (29.0)
I couldn’t afford the cost of visits or treatments	861 (27.3)	231 (25.9)	419 (25.4)	96 (31.1)*	115 (37.1)**
I thought it would be meaningless if it couldn’t be cured completely	848 (26.8)	224 (25.1)	409 (24.8)	98 (31.7)*	117 (37.7)**
I thought I didn’t need to go to the hospital for just a headache	838 (26.5)	226 (25.3)	430 (26.1)	85 (27.5)	97 (31.3)*
I didn’t know which medical institution or doctor to consult	735 (23.3)	210 (23.5)	358 (21.7)	82 (26.5)	85 (27.4)
Even after consulting, I was only prescribed medication similar to OTC headache medicine	726 (23.0)	149 (16.7)	390 (23.7)*	91 (29.4)**	96 (31.0)**
I thought the doctor would say it wasn’t a big deal	715 (22.6)	197 (22.1)	368 (22.3)	73 (23.6)	77 (24.8)
I didn’t have time	700 (22.2)	163 (18.3)	378 (23.0)	72 (23.3)*	87 (28.1)*
I thought my headache wasn’t a big deal	656 (20.8)	192 (21.5)	342 (20.8)	72 (23.3)	50 (16.1)
I thought there wouldn’t be better treatments or remedies than what I was already using	595 (18.8)	148 (16.6)	283 (17.2)	70 (22.7)*	94 (30.3)**
I thought the doctor wouldn’t understand the severity or anxiety of my headache	535 (16.9)	159 (17.8)	216 (13.1)	68 (22.0)	92 (29.7)**
The symptoms occurred infrequently	520 (16.5)	165 (18.5)	293 (17.8)	36 (11.7)	26 (8.4)
The symptoms were mild even when they occurred	363 (11.5)	97 (10.9)	211 (12.8)	32 (10.4)	23 (7.4)
I was afraid of being diagnosed with a serious illness	325 (10.3)	74 (8.3)	174 (10.6)	39 (12.6)	38 (12.3)
The impact of COVID-19	323 (10.2)	71 (8.0)	173 (10.5)	38 (12.3)	41 (13.2)*
I didn’t want to undergo tests	276 (8.7)	61 (6.8)	138 (8.4)	32 (10.4)	45 (14.5)*
I didn’t want to be diagnosed with migraines or chronic headaches	204 (6.5)	43 (4.8)	97 (5.9)	33 (10.7)*	31 (10.0)*
Other reasons	119 (3.8)	68 (7.6)	39 (2.4)	4 (1.3)	8 (2.6)

* and ** indicate items with ≥ 5% and ≥ 10% higher proportions versus the 0 monthly days of OTC use subgroup, respectively

^1^ These respondents have experienced consultations with a doctor for their headaches. ^2^ Only includes respondents who hesitated to visit a doctor. **Abbreviations**: COVID – Coronavirus Disease, n – Sample size, OTC – Over-the-counter

Among 4605 respondents who answered that they communicated with doctors in the past year, 14.6% spoke about OTC drug use and 12.5% spoke about new treatments in their consultations with the doctors. This proportion increased to 27.2% and 19.1% in respondents with ≥ 10 monthly days of OTC use.

## Discussion

This real-world analysis of OTC drug use in Japanese respondents with migraine described the challenges they potentially face in accessing appropriate medical management. The study highlighted that nearly three-fourths of the respondents with migraine used OTC drugs in the past year, irrespective of the number of MHDs. Most respondents (79.0%) used OTC drugs more than prescribed acute drugs, although this proportion reduced with an increase in the number of MHDs. More than one-third of the respondents answered that they would usually use OTC drugs in addition to prescribed acute drugs when they had migraine attacks. This may increase the risk of MOH unless a doctor is aware of the OTC drug use and manages the prescribed acute drug use accordingly. Compared with the overall population, the use of OTC drugs was less among respondents who consulted doctors for migraine in the past year; however, the overall trend regarding frequent OTC drug use was still observed. Only 14.6% of respondents with migraine discussed OTC drugs with their doctors; access and awareness about migraine-specific prescription drugs were also limited.

The high prevalence of OTC drug use observed in this study (72.7%) is consistent with previous OVERCOME studies from Japan (75.2%) [4], the EU (70.5%) [23], and the US (81.8%) [18]. In the current study, the prevalence of OTC drug use was also high (65.6%) among respondents who consulted doctors over the past year. NSAIDs and triptans were the most commonly prescribed acute drugs in the current study, in line with previous Japanese studies [4, 10].

Compared with the overall population, in the subpopulation of respondents who consulted doctors for migraine in the past year, prescribed acute and preventive drug use increased, while OTC drug use decreased. This is expected, as prescribed acute and preventive drugs will only be dispensed with a doctor’s prescription. Although 62.3% of this subpopulation would usually use prescribed drugs for migraine attacks, 31.3% of them would use OTC drugs in addition to prescribed drugs, similar to a previous Japanese real-world study (31.8%) [24]. The consulting doctors may not be aware of such concomitant use of OTC and prescription drugs. More specifically, in the current study, among respondents who would usually use prescribed NSAIDs, more than one-third would also use NSAID-containing OTC drugs. This is concerning as such medication overuse may lead to MOH [9].

When the respondents were grouped by monthly days of OTC drug use, there was a notable trend: respondents with ≥ 10 monthly days of OTC drug use were diagnosed with migraine the earliest in life (mean age 25.1 years). The previous report from the OVERCOME (Japan) 2nd study (i.e., the current study), reported that OTC drugs were the first medications respondents took for their headaches (at a mean age of 19.4 years) after they started experiencing headaches (at a mean age of 17.8 years) [14]. This is likely due to the easier access for OTC drugs compared to prescription drugs. Overall, these results suggest that long-term use of OTC medications by people who started experiencing migraine at a younger age may have led to frequent use of those medications by the time of the survey i.e., later in life. Moreover, respondents with ≥ 5 monthly days of OTC drug use had numerically more MHDs and greater migraine-related burden than respondents with ≤ 4 monthly days of OTC drug use, suggesting that current medications may be inadequate to meet the needs of people with severe migraine. We also report this in Fig. 2, where both prescribed acute drug use and OTC drug use tended to be greater among respondents with more MHDs.

Since only 14.6% of respondents with migraine and 27.2% of those with ≥ 10 monthly days of OTC drug use discussed OTC drug use with doctors in the current study, doctors might be unaware of concurrent prescribed acute and OTC drug use. Additionally, claims databases, which are increasingly commonly used for medical research, also do not capture OTC drug use [10]. These highlight the challenges doctors face during consultations in accurately capturing OTC drug use by the patients and underscore the importance of careful doctor-patient communication to understand the status of patients concerning their drug treatment. Furthermore, ≥ 73.1% of respondents were unaware of migraine-specific drugs, probably because they did not communicate about new treatments with their doctors. In fact, only 12.5% of respondents spoke about new treatments with their doctors. This can partly be due to the unfamiliarity of migraine-specific drugs among consulting doctors, especially general practitioners [25, 26]. Since people with migraine mostly consult general practitioners in the real world [4, 18, 23], non-specialist doctors also play an important role in the medical management of migraine. In our previous report from the OVERCOME (Japan) 2nd study, the respondents received migraine-specific drugs such as triptans more than a decade after headache onset [14]. This indicates the need for non-specialist doctors such as general practitioners to be educated about migraine and consider referral to a specialist if required [27, 28], thus reducing the time taken for treatment and decreasing patient burden [29].

Approximately half of the respondents with ≥ 5 monthly days of OTC drug use hesitated to visit doctors because they thought they could handle the headaches with OTC drugs. Other reasons for hesitation, such as “I thought I didn’t need to go to the hospital for just a headache” and “I thought the doctor wouldn’t understand the severity or anxiety of my headache,” which were highest in the ≥ 10 monthly days of OTC drug use subgroup, have also been reported before [30–32]. Reasons for hesitation such as ‘Even after consulting, I was only prescribed medication similar to OTC headache medicine’ and ‘I thought there wouldn’t be better treatments or remedies than what I was already using’ suggest that approximately 30% of respondents in the ≥ 10 monthly days of OTC drug use subgroup may have been unaware of the potential benefit of migraine-specific drugs. Despite such hesitations, people in the ≥ 10 monthly days of OTC drug use subgroup experienced an average of 11.5 MHDs. It is possible that the Japanese cultural belief in ‘*gaman*’, i.e., ‘perseverance,’ ‘tolerance,’ or ‘self-denial,’ leads them to fight through the migraine-related burden in their daily lives and deprioritize their migraine management as much as possible [30]. However, poor migraine management can increase absenteeism and presenteeism [4, 30], negatively impact work productivity [4, 30], and add to migraine-related costs [33]. To prevent this burden, patient education is necessary. This involves increasing awareness about the risks of not seeking appropriate medical care (such as MOH and progression to CM), informing patients about the disease and cutting-edge treatment options, counseling them about realistic treatment expectations, and providing guidance on accessing suitable treatment centers [31, 34, 35].

The prevalence rate of probable MOH in the current study (2.6%) was consistent with that of previous Japanese studies (2.3–3.7%) [7, 10]. By definition, MOH can only be diagnosed in individuals with ≥ 15 MHDs [9], however, medication overuse per ICHD-3 criteria [9] can occur in individuals with < 15 MHDs too. Real-world studies from the EU [36] and the US [37] reported a medication overuse prevalence of 10.7–17.7%; respondents had an average of 11.2–12.1 MHDs. Furthermore, respondents with medication overuse had greater disease burden and healthcare resource utilization than those without medication overuse [36, 37]. The current study did not assess medication overuse prevalence; however, 29.6% of respondents with ≥ 10 monthly days of OTC drug use had probable MOH, suggesting that medication overuse, especially in this subgroup, may be high.

Medication overuse is associated with the risk of migraine progression from episodic migraine to CM [38–40]. Hence, it is essential for doctors to actively communicate with patients about OTC drug usage, educate them about medication overuse and MOH, and provide appropriate medical intervention if required [41]. Among new migraine medications, CGRP mAbs reduced MHDs in patients with MOH by 22.7% after a month’s treatment [42]. This suggests that CGRP mAbs may be a viable treatment option for individuals with MOH. However, treatment with CGRP mAbs is expensive [43], so patients need to meet certain criteria to receive reimbursement before receiving treatment. In Japan, CGRP mAbs are recommended for and reimbursed for patients who experience ≥ 4 monthly migraine days and ≥ 1 oral preventive treatment failure [44]. Basically, patients have to pay 30% of the cost and the rest is covered by Japan’s national health insurance [44]. Reimbursement criteria also vary widely in Europe [45]. However, the criteria are much more stringent in the US [46], and the insurance coverage depends on the patient’s insurance plan and the insurance company’s reimbursement criteria [44]. Issues such as delays and denials in obtaining prior authorization, non-medical switching, and lack of coverage for combination therapy also impact patient access to appropriate treatment [47]. A recent change in German reimbursement policy allowed erenumab prescriptions for patients with migraine who failed one preventive medication [48]. This led to a significantly greater reduction in MHD and an increase in ≥ 50% response rate compared with patients treated per the previous policy. Similar studies in other countries could generate evidence supporting early initiation of CGRP mAbs in the course of migraine progression and thus increase patient access to these medications.

### Strengths and limitations

Danno et al. [14] describes the strengths and limitations of the OVERCOME (Japan) 2nd study. Describing the medical management of migraines and patient attitudes towards migraines by monthly use of OTC drug subgroups is a major strength of the current analysis because capturing this information through medical consultations is difficult for doctors. Such data may be helpful to alter migraine management based on an individual’s monthly use of OTC drugs. This analysis has some limitations too. Due to its online nature, the study only included participants with internet access. To assess the monthly use of drugs, days of individual drug use were summed up for each drug class (prescribed acute or OTC drugs), so we may have overestimated the number of days when multiple drugs were used in combination on the same day. Respondents self-reported the data so we cannot rule out recall bias. The current survey included similar or common questions as the previous OVERCOME (Japan) study in 2020 [4], but some questions were newly created for the current survey or adapted from previous OVERCOME studies [17, 18]. Such questions may not have been validated in the Japanese population.

## Conclusion

The current analysis of the OVERCOME (Japan) 2nd study shows that OTC drug use is common among respondents with migraine, regardless of the number of MHDs, and even among those who have consulted doctors and are on prescribed acute drugs. Some respondents even use OTC drugs more frequently than or along with prescribed acute drugs. OTC drug use is not commonly discussed during consultations. Moreover, the majority of the respondents, even those who were consulting doctors or with frequent OTC drug use, did not have access to or awareness of migraine-specific drugs. Respondents also did not prioritize migraine management. Therefore, to prevent MOH, doctors should communicate with patients about OTC drug use, treat them with appropriate prescription drugs, and provide adequate information and medical intervention.

## Electronic supplementary material

Below is the link to the electronic supplementary material.

Supplementary Material 1

## Data Availability

The datasets generated and/or analyzed during the current study are available at Eli Lilly Japan K.K on reasonable request.

## Abbreviations

CGRP mAbs: Calcitonin Gene-related Peptide Monoclonal Antibodies
CM: Chronic Migraine
HIT: 6-Headache Impact Test-6
ICHD: 3-International Classification of Headache Disorders-3rd edition
MHD: Monthly Headache Days
MIBS: 4-Migraine Interictal Burden Scale-4
MIDAS: Migraine Disability Assessment Scale
MOH: Medication Overuse Headache
OTC: Over-the-counter
OVERCOME: ObserVational survey of the Epidemiology, tReatment, and Care Of MigrainE
PRO: Patient-reported Outcomes
