# Supplementary material for: Real-world use of over-the-counter medications by patients with migraine in Japan: results from the OVERCOME (Japan) 2nd study
Source: J Headache Pain. 2025 May 7;26(1):107. doi: 10.1186/s10194-025-02046-8 (PMC12056991; doi:10.1186/s10194-025-02046-8)
Supplement: Supplementary file 1 — Supplementary Material 1 [file 10194_2025_2046_MOESM1_ESM.docx]

**Additional files**

**Additional file 1: Patient-reported outcomes**

| **PRO** | **Evaluation** | **Scoring** | **Grading** |
| --- | --- | --- | --- |
| **MIDAS [19]** | Quantifies headache-related disability over a 3-month period. It  measures the number of days missed or with reduced productivity at work, home, or social events. | The total score is a summation of five items (scored 0–90); higher values indicate greater disability. | Grade I (0–5) – little or no disability, Grade II (6–10) – mild disability, Grade III (11–20) – moderate disability, and Grade IV (21+) – severe disability. |
| **MIDAS additional question on pain severity [19]** | Patients self-report average headache pain severity on an 11-point numerical rating scale. | 0 (no pain at all) to 10 (pain as bad as it could be). | - |
| **HIT-6 [20]** | A 6-item scale that measures the impact of headaches on activities in work, school, home, and society. | Scored 36–78; higher scores indicate greater interference in daily life. | Little-to-no impact (36–49), Moderate impact (50–55), Substantial impact (56–59), and Severe impact (60–78). |
| **MIBS-4 [21, 22]** | A 4-item instrument that measures headache-related burden in the time between attacks. The items address disruption at work and school, diminished family and social life, difficulty planning, and emotional difficulty. | The total score range is 0–12. | Level of interictal burden is categorized as None (0), Mild (1–2), Moderate (3–4), and Severe (5+). |

**Abbreviations:** HIT-6 – Headache Impact Test-6, MIBS-4 – Migraine Interictal Burden Scale-4, MIDAS – Migraine Disability Assessment Scale, PRO – Patient-Reported Outcome

**Additional file 2: OTC drugs available in Japan [49]**

| **Brand name** | **Ingredients** |
| --- | --- |
| EVE A tablets/EX/Quick/DX | Ibuprofen + other ingredients |
| Kerorin | Aspirin + other ingredients |
| Sedes High/High G/New Sedes tablets | Acetaminophen + NSAIDs |
| Tylenol A | Acetaminophen only |
| Dipacio IPa | Ibuprofen + other ingredients |
| Naron Ace T | Ibuprofen + other NSAIDs |
| Naron tablets | Acetaminophen + NSAIDs |
| Norshin | Acetaminophen + NSAIDs |
| Norshin Pure | Ibuprofen + other ingredients |
| Hakkiri Ace a | Acetaminophen + NSAIDs |
| Bufferin A | Aspirin + other ingredients |
| Bufferin Premium/Premium DX | Ibuprofen + Acetaminophen |
| Bufferin Luna i | Ibuprofen + Acetaminophen |
| Bufferin Luna J | Acetaminophen only |
| Meridon EV tablets | Ibuprofen + other ingredients |
| Lackle | Acetaminophen only |
| Ringl IB/α200 | Ibuprofen only |
| Loxonin S | Loxoprofen only |
| Loxonin S Quick/Plus/Premium | Loxoprofen + other ingredients |
| Loxoprofen Sodium Tablets KUNIHIRO | Loxoprofen only |
| Others |  |

**Abbreviation:** NSAID – Non-Steroidal Anti-Inflammatory Drug

**Additional file 3: Subtypes of probable medication overuse headache**

| **Subtypes by drug class** | **Probable MOH (n = 503)** |
| --- | --- |
| Combined analgesics-overuse headache, n (%) | 284 (56.5) |
| NSAIDs-overuse headache, n (%) | 174 (34.6) |
| Triptan-overuse headache, n (%) | 57 (11.3) |
| Acetaminophen-overuse headache, n (%) | 38 (7.6) |
| Opioid-overuse headache, n (%) | 24 (4.8) |
| Ergotamine-overuse headache, n (%) | 3 (0.6) |

**Abbreviations:** MOH – Medication Overuse Headache, n – Sample size, NSAID – Non-Steroidal Anti-Inflammatory Drug

**Additional file 4: Demographic characteristics, clinical characteristics, and patient-reported outcomes (by monthly days of OTC use)**

|  | **0 days**  **(n = 6806)** | **1–4 days**  **(n = 10,362)** | **5–9 days**  **(n = 1267)** | **≥10 days**  **(n = 1155)** |
| --- | --- | --- | --- | --- |
| **Age (years), mean (SD)** | 40.9 (13.8) | 40.2 (12.9) | 40.0 (12.3) | 41.2 (12.1) |
| **Female, n (%)** | 4468 (65.6) | 7283 (70.3) | 921 (72.7) | 814 (70.5) |
| **Married, n (%)** | 2901 (42.6) | 4927 (47.5) | 571 (45.1) | 482 (41.7) |
| **Employed, n (%)** | 4034 (59.3) | 7064 (68.2) | 856 (67.6) | 770 (66.7) |
| **Diagnosis of migraine by a physician, n (%)** | 3227 (47.4) | 4550 (43.9) | 663 (52.3) | 641 (55.5) |
| Age at migraine diagnosis, mean (SD) | 28.4 (12.0) | 26.2 (11.0) | 26.5 (10.9) | 25.1 (10.4) |
| **Met ICHD-3 criteria** |  |  |  |  |
| Met ICHD-3 criteria and diagnosed as migraine | 5697 (83.7) | 8770 (84.6) | 1121 (88.5) | 1039 (90.0) |
| **Average MHDs in the past 90 days, mean (SD)** | 3.2 (5.3) | 2.5 (3.4) | 6.1 (5.0) | 11.5 (9.0) |
| **Pain severity, mean (SD)** | 5.4 (2.3) | 5.7 (1.9) | 6.5 (1.6) | 6.8 (1.6) |
| **MIDAS score, mean (SD)** | 8.8 (21.1) | 7.7 (15.2) | 18.3 (25.8) | 29.9 (38.8) |
| **MIDAS grade, n (%)** |  |  |  |  |
| Grade I (0–5) | 4640 (68.7) | 6614 (63.9) | 473 (37.7) | 383 (34.2) |
| Grade II (6–10) | 744 (11.0) | 1612 (15.6) | 192 (15.3) | 111 (9.9) |
| Grade III (11–20) | 642 (9.5) | 1205 (11.6) | 233 (18.6) | 140 (12.5) |
| Grade IV (21+) | 724 (10.7) | 916 (8.9) | 358 (28.5) | 487 (43.4) |
| **HIT-6 score, mean (SD)** | 58.4 (8.5) | 59.7 (7.1) | 62.9 (6.5) | 64.4 (7.0) |
| **HIT-6 grade, n (%)** |  |  |  |  |
| Little-to-no impact (36–49) | 1088 (16.0) | 852 (8.2) | 35 (2.8) | 29 (2.5) |
| Moderate impact (50–55) | 1253 (18.4) | 1768 (17.1) | 95 (7.5) | 80 (6.9) |
| Substantial impact (56–59) | 1054 (15.5) | 1938 (18.7) | 179 (14.1) | 112 (9.7) |
| Severe impact (60–78) | 3411 (50.1) | 5804 (56.0) | 958 (75.6) | 934 (80.9) |
| **MIBS-4 score, mean (SD)** | 3.0 (3.6) | 3.1 (3.5) | 4.3 (3.8) | 4.9 (4.0) |
| **MIBS-4 level of interictal burden, n (%)** |  |  |  |  |
| None (0) | 3172 (46.6) | 4402 (42.5) | 353 (27.9) | 289 (25.0) |
| Mild (1–2) | 650 (9.6) | 1227 (11.8) | 153 (12.1) | 122 (10.6) |
| Moderate (3–4) | 949 (13.9) | 1563 (15.1) | 203 (16.0) | 171 (14.8) |
| Severe (5+) | 2035 (29.9) | 3170 (30.6) | 558 (44.0) | 573 (49.6) |
| **Ever visited a doctor, n (%)** | 4178 (61.4) | 5925 (57.2) | 833 (65.7) | 822 (71.2) |
| Over the past year | 2355 (34.6) | 2508 (24.2) | 389 (30.7) | 432 (37.4) |
| **Probable MOH, n (%)** | 127 (1.9) | 30 (0.3) | 4 (0.3) | 342 (29.6) |

During data cleaning, respondents with inappropriate responses for MIDAS questions, i.e., >90 days of disability due to migraine in a 90-day period, were excluded. **Abbreviations:** HIT-6 – Headache Impact Test-6, ICHD-3 - International Classification of Headache Disorders – 3^rd^ edition, MHD – Monthly Headache Days, MIBS – Migraine Interictal Burden Scale-4, MIDAS – Migraine Disability Assessment Scale, MOH – Medication Overuse Headache, N – Total population size, n – Sample size, OTC – Over-the-counter, SD – Standard Deviation
